# The Therapeutic Potential of Targeting NIK in B Cell Malignancies

**DOI:** 10.3389/fimmu.2022.930986

**Published:** 2022-07-12

**Authors:** Marco V. Haselager, Eric Eldering

**Affiliations:** ^1^ Department of Experimental Immunology, Amsterdam University Medical Center, Amsterdam, Netherlands; ^2^ Lymphoma and Myeloma Center Amsterdam, Lymphoma and Myeloma Center Amsterdam, Amsterdam, Netherlands; ^3^ Cancer Center Amsterdam, Cancer Immunology, Amsterdam, Netherlands; ^4^ Amsterdam Institute for Infection and Immunity, Cancer Immunology, Amsterdam, Netherlands

**Keywords:** NIK, therapeutic targets, B cell malignancies, small molecule inhibitors, *in vitro*, *in vivo*

## Abstract

NF-κB-inducing kinase (NIK) is a key player in non-canonical NF-κB signaling, involved in several fundamental cellular processes, and is crucial for B cell function and development. In response to certain signals and ligands, such as CD40, BAFF and lymphotoxin-β activation, NIK protein stabilization and subsequent NF-κB activation is achieved. Overexpression or overactivation of NIK is associated with several malignancies, including activating mutations in multiple myeloma (MM) and gain-of-function in MALT lymphoma as a result of post-translational modifications. Consequently, drug discovery studies are devoted to pharmacologic modulation of NIK and development of specific novel small molecule inhibitors. However, disease-specific *in vitro* and *in vivo* studies investigating NIK inhibition are as of yet lacking, and clinical trials with NIK inhibitors remain to be initiated. In order to bridge the gap between bench and bedside, this review first briefly summarizes our current knowledge on NIK activation, functional activity and stability. Secondly, we compare current inhibitors targeting NIK based on efficacy and specificity, and provide a future perspective on the therapeutic potential of NIK inhibition in B cell malignancies.

## Introduction

**Figure 1:**
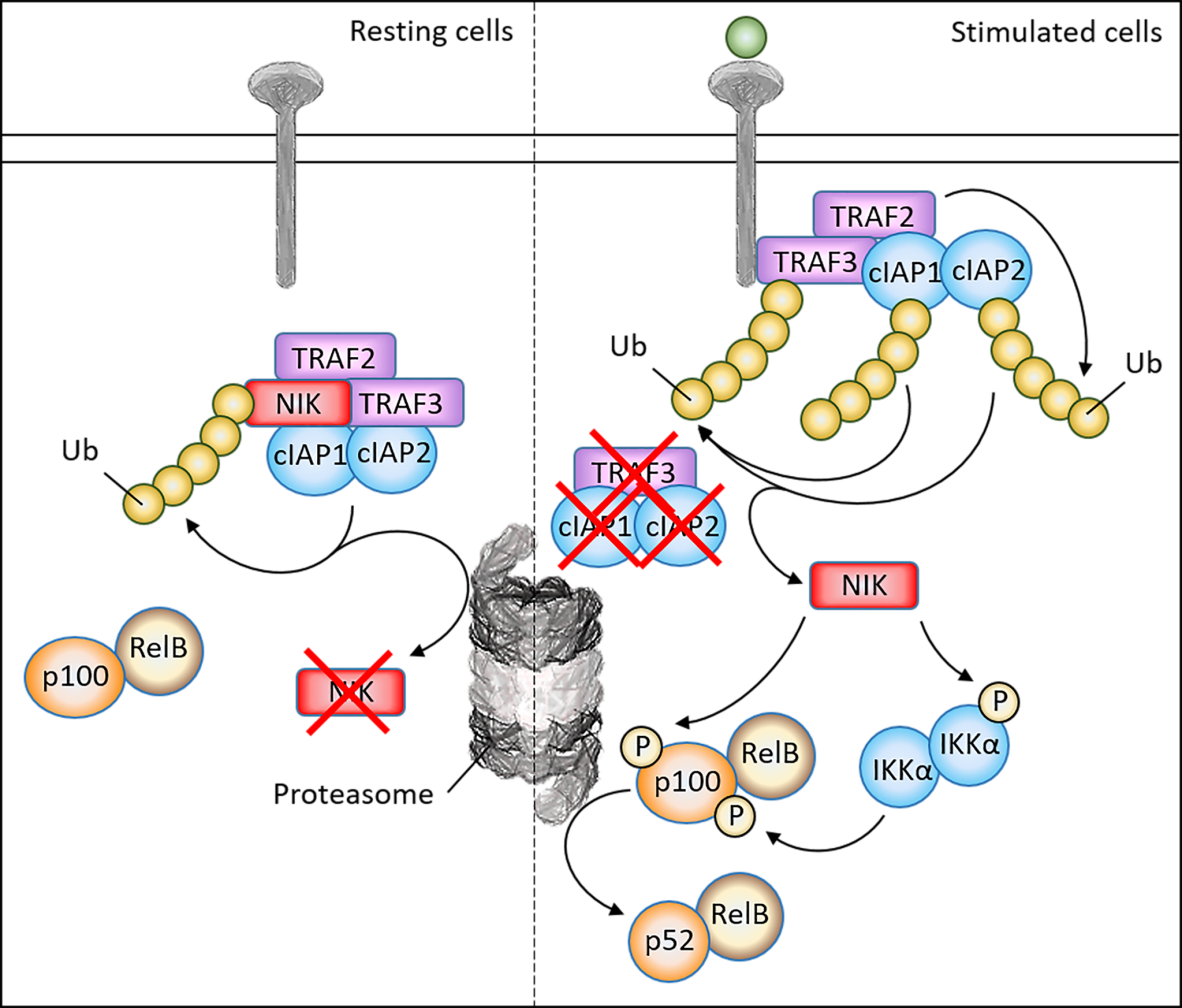
Schematic overview of non-canonical signaling pathway and NIK regulatory mechanisms. In resting cells, NIK forms a complex with TRAF2/3 and cIAP1/2, which leads to the continuous ubiquitination and proteasomal degradation of NIK. Upon ligand binding, TRAF3 is recruited to the receptor, where TRAF2 induces ubiquitination of cIAP1/2 which in turn induces ubiquitination of TRAF3. As a result, TRAF3 is degraded and NIK is stabilized. After NIK stabilization, NIK is able to phosphorylate the IKKα complex to induce activation. After direct phosphorylation of the precursor p100 by both NIK and the IKKα complex, p100 will undergo proteasomal processing into the active subunit p52, which together with RelB may translocate to the nucleus to activate target genes.

Nuclear factor kappa B (NF-κB) comprises a family of transcription factors that regulate a wide array of genes involved in inflammation, immunity, differentiation, proliferation and cell survival. As NF-κB is ubiquitously expressed, dysregulation of NF-κB is an important contributor to both the development of various cancers and their progression (1). NF-κB signaling can be distinguished into two distinct pathways, the canonical and non-canonical pathway. NF-κB inducing kinase (NIK), which is encoded by the gene *MAP3K14*, has been identified as the central kinase controlling non-canonical NF-κB activation (**Figure 1**). The non-canonical NF-κB pathway can be activated upon stimulation of a subset of upstream activators, including activation of the BAFF receptor (BAFF-R), CD40, receptor activator of NF-κB (RANK) or the lymphotoxin beta receptor (LT-βR) (2, 3). Stimulation of these receptors will recruit TRAF2/3 to the receptor, resulting in the accumulation of NIK protein levels. NIK is able to directly phosphorylate the precursor p100 at Ser866/870 and induce phosphorylation of IKKα homodimers at Ser176/180 (4–6). In turn, IKKα complexes will phosphorylate p100 at Ser872, which together with Ser866/870 phosphorylation by NIK is required to result in the proteasomal processing of p100 into p52 (7, 8). Whereas p100/RelB dimers are sequestered in the cytoplasm, p52/RelB dimers are free to translocate to the nucleus in order to activate target genes. Because of this role of NIK in non-canonical NF-κB signaling, NIK is crucial in regulating immunity and inflammation, where loss of NIK is associated with severe immune defects whereas NIK overexpression is observed in inflammatory diseases and malignancies (9). For this reason, targeting of NIK and the non-canonical NF-κB pathway may have therapeutic potential in various diseases.

## NIK Activity

NIK is a kinase that does not require phosphorylation in order to become activated (10). It was previously thought that phosphorylation of Thr559 would result in NIK activation (11). However, subsequent studies could not identify phosphorylation of Thr559 and in addition showed that mutation of this site results in similar kinase activity (12). Structural studies revealed a constitutively active conformation of NIK (13). This suggests that NIK is predominantly regulated at the protein level by inducing its degradation as previously described. Several proteins have been suggested to regulate the activity of NIK rather than its stability, such as TRAFs and NIK-associated protein encoded by the *TNAP* gene, and Phosphatidylethanolamine binding protein 1 encoded by the *PEBP1* gene, though their mechanism of NIK inactivation has not been well elucidated (14, 15).

## NIK Regulation

The basal level of NIK protein is very low in resting cells yet NIK is stabilized after stimulation of upstream receptors such as CD40 or BAFF-R after which the half-life of NIK increases to about 3 hours (16). The accumulation of NIK protein levels is the key event which permits the activation of the alternative NF-κB pathway. As a consequence, activation of non-canonical NF-κB dimers takes more time compared to canonical NF-κB dimers, due to the requirement for the accumulation of NIK by *de novo* protein synthesis (17).

There has been a lot of investigation into factors that regulate NIK degradation and stability, which is important in order to design potential therapies targeting this key kinase. Arguably the most important proteins involved in the regulation of NIK are the TNF receptor associated factor (TRAF) proteins and cellular inhibitor of apoptosis (cIAP) proteins. In resting cells, NIK forms a complex with TRAF2/3 and cIAP1/2, which leads to the continuous ubiquitination and proteasomal degradation of NIK. Upon ligand binding, TRAF3 is recruited to the respective TNFR, where TRAF2 induces ubiquitination of cIAP1/2 which in turn induces ubiquitination of TRAF3. As a result, TRAF3 is degraded and NIK is stabilized (18).

In addition to constitutive regulation by the TRAF-cIAP complex, it has been demonstrated that NIK is also subject to negative feedback regulation (19). IKKα phosphorylates NIK at Ser809/812/815 to induce NIK proteasomal degradation thereby limiting its own activation. Phosphorylation of NIK by TBK1 at Ser862 has also been identified to destabilize and induce degradation of NIK, in a TRAF3-independent manner (20, 21). Additionally, expression of NEMO was found to suppress NIK protein levels, suggesting a mechanism for the canonical NF-κB signaling pathway to limit activity of non-canonical NF-κB activation (22).

In addition to these well-described mechanisms, there are several additional factors that are thought to be involved in the regulation of NIK. One such example is Hsp90, which has been proposed to control NIK protein folding and stabilization (23). Inhibition of Hsp90 disrupts its interaction with NIK, resulting in NIK degradation independent of ubiquitination and which is mediated by autophagy. Moreover, the protein CHIP, encoded by the gene *STUB1*, is shown to be able to directly bind NIK and thereby induce NIK degradation and inhibit non-canonical NF-κB activity (24). Although CHIP contains ubiquitin E3 ligase activity, this is not actually required for the ubiquitination of NIK. This suggests that many more NIK-interacting proteins could serve as adaptor proteins coordinating the regulation of NIK, possibly by functioning in concordance with the TRAF/cIAP machinery. However, a few proteins have also been reported to directly induce ubiquitination of NIK independent of TRAF/cIAP, like the E3 ligase CRL4 (25).

NIK is encoded by the *MAP3K14* gene which requires active transcription before NIK can be regulated at the protein level. Importantly, NIK seems to positively regulate its own transcription (26), creating a positive feedback loop when TRAF3 is degraded. At the same time, NIK may shape a transcriptional negative feedback loop, as cells with constitutively active NIK mutants show increased TRAF2 and TRAF3 mRNA expression (27). Although not much is known about the transcriptional regulation of *MAP3K14*, an increasing number of observations point towards a complex transcriptional regulation. It was shown that *MAP3K14* mRNA may be targeted by nonsense-mediated decay to prevent NIK overactivity which is disrupted in myofibroblastic tumors (28). Additionally, there is evidence of regulation of *MAP3K14 via* various microRNAs (29–33).

## Role of NIK in B Cell Malignancies

A tumor can establish elevated NF-κB activity by either extrinsic or intrinsic factors. BAFF-R, CD40, RANK and LT-βR signaling play a role in several B cell malignancies associated with increased non-canonical NF-κB activation (34–40). Whereas in some B cell malignancies this is established by the microenvironment, others may acquire mutations that promote non-canonical NF-κB signaling. For example, up to 20% of MM cases display constitutive non-canonical NF-κB signaling as a result from genetic aberrations of NF-κB regulatory genes. Bi-allelic deletions observed in MM include TRAF2, *TRAF3*, *BIRC2* (encoding cIAP1) and *BIRC3* (encoding cIAP2), which lead to their inactivation and subsequent activation of NIK (41). Gene expression profiling cohorts also indicate overexpression of *MAP3K14* itself, indicating increased transcription of NIK. *TRAF3* deletions and *MAP3K14* gains are also frequently observed in Hodgkin lymphoma (42). Additionally, *MAP3K14* translocation to the *IGH* or *IGL* locus has been found in MM patients and leads to a significant increase of NIK expression. One specific *MAP3K14* translocation to the *EFTUD2* gene results in an EFTUD2-NIK fusion protein which results in a stable form of NIK that lacks the binding region to TRAF3 (43). Other overexpressed genes include *LTBR*, *CD40* and *TNFRSF13B*, which all encode for upstream activators of non-canonical signaling and thus prime cells for NIK activation, respectively. Additionally, *TRAF3* mutations are observed resulting in truncated TRAF3 protein that is no longer able to bind and regulate NIK. The most common cytogenetic abnormality in MALT lymphoma is the chromosomal translocation t(11;18)(q21;q21) which creates the API2-MALT1 fusion protein (44). This fusion protein cleaves several substrates, including NIK at Arg325, which generates a stable NIK fragment that is catalytically active, resulting in constitutive non-canonical NF-κB signaling. 5-10% of acute myeloid leukemias harbor genomic rearrangements resulting in the MLL-AF9 fusion gene, associated with stabilized NIK and constitutive non-canonical NF-κB activation, thereby exerting anti-apoptotic effects (45). Furthermore, NIK is overexpressed in anaplastic large cell lymphoma, adult T cell leukemia and peripheral T cell lymphoma (46–48). In mantle cell lymphoma, ibrutinib-resistant patients show recurrent mutations in *TRAF2* or *BIRC3* associated with constitutive non-canonical NF-κB signaling and a complete dependence on NIK (49). Therefore, genomic profiling reveals NIK to be a valuable therapeutic target in several B cell malignancies and NIK may become even more important in the case of resistance or non-responsiveness to B cell receptor inhibitors.

## Genetic Targeting of NIK

As most cells under physiological conditions do not have nor require abundant NIK expression and NIK becomes stabilized in pathological contexts, NIK is a potential clinical target. Notably, NIK is not essential for the activation of the canonical NF-κB pathway which may translate to limited side effects upon inhibition *in vivo*. The fact that NIK is overexpressed and/or hyperactivated in many B cell malignancies including other cancers, suggests that inhibition of NIK has clear clinical potential. Yet, most preclinical studies have relied on genetic targeting of NIK. NIK knockout mice and alymphoplasia (aly/aly) mice deficient for NIK suffer from a lack of lymph nodes, Peyer’s patches and abnormal spleen architecture (50–52). NIK-deficient mice have impaired germinal center formation, somatic hypermutation and antigen-specific antibody production (51, 53, 54), which is consistent with biallelic mutation of NIK in B cell lymphopenia patients who have decreased numbers of class-switched B cells and hypogammaglobulinemia (55). On the other hand, NIK deficiency has little effect on thymocyte development (56), though NIK is necessary for Th17 differentiation (57). Moreover, NIK is required for antigen-specific signaling in effector and memory T cells (58). Additionally, NIK knockout and aly/aly mice have fewer regulatory T cells (59). NIK knockout results in defective LT-βR signaling and conditional deletion of NIK in adult mice results in defects in B cell activation and survival as well as defective signaling upon BAFF, anti-CD40 or anti-IgM stimulation (53, 60), which may indicate great promise of NIK inhibition in B cell malignancies that are largely dependent on microenvironmental signals, such as chronic lymphocytic leukemia (CLL).

## Role of NIK in Crosstalk Between Canonical and Non-Canonical NF-κB Signaling

Ideally, a specific NF-κB inhibitor should only target the corresponding canonical or non-canonical pathway without effects on other signaling pathways. Despite the seemingly clear delineation of canonical versus non-canonical NF-κB signaling, there appears to be crosstalk between these two pathways. For example, it has been demonstrated that sustained high NIK protein levels resulted in enhanced canonical NF-κB activity in response to a variety of stimuli (61). Similar findings were shown in MM where NIK overexpression triggered constitutive non-canonical signaling and often activation of the canonical NF-κB pathway as well (41, 43, 62). It may be the case that NIK overexpression enables binding of NIK to additional NF-κB regulatory proteins that have a lower binding affinity, such as IKKβ in the canonical pathway. In support, NIK-dependent activation of canonical NF-κB signaling has been shown *via* the interaction of NIK with the IKKα/IKKβ complex in response to LT-βR ligation (63). Importantly, p100 contains inhibitory functions by sequestering both canonical and non-canonical NF-κB dimers in the cytoplasm (64). In this way, NIK may promote both canonical and non-canonical NF-κB activation as a result of the processing of p100 into p52 and therefore targeting of NIK may inhibit both NF-κB pathways. The ability of NIK to contribute to both NF-κB pathways is a probable cause why its dysregulation is associated with malignancies, especially in B cells which are reliant on continuous signaling through both pathways for cell proliferation and survival. On one hand, this makes NIK an excellent therapeutic target as it can modulate both NF-κB signaling pathways, but on the other hand, targeting of NIK may result in unwanted toxicities or side effects when selective inhibition of non-canonical NF-κB is preferable. The exact conditions and mechanisms on when and how NIK contributes to canonical NF-κB signaling are important to understand. If the contribution of NIK to canonical NF-κB is restricted to its overexpression, then NIK will indeed be a highly interesting target for therapy as its inhibition should have limited side effects on healthy cells yet be detrimental to cancer cells (65). If NIK is implicated in conventional canonical NF-κB activation, targeting of NIK may cause severe toxicities. Although NIK-deficient mouse models display clear impairments in adaptive immunity, *de novo* targeting of NIK may have a different impact compared to NIK deficiency during development. Despite the fact that no NIK inhibitor has been clinically approved yet, a number of small molecule inhibitors targeting NIK have been developed. In the next section, we provide an overview and comparison of NIK inhibitors published in literature based on efficacy and specificity.

## Overview of *In Vitro* NIK Inhibitors

Though NIK seems a promising potential therapeutic target, studies on NIK inhibitors were limited due to missing structural data. Some *in vitro* studies have shown promising results with NIK inhibition in Hodgkin lymphoma (66) and mantle cell lymphoma (MCL) (49), yet these studies did apply inhibitors designed before publication of the crystal structure of NIK in 2012 (12, 13). Furthermore, some studies report NIK inhibition after application of pan-kinase inhibitors or naturally occurring compounds that inhibit a wide spectrum of kinases (67–69). Virtually all inhibitors designed to specifically target NIK are small molecule inhibitors aimed at binding the catalytic ATP-binding site, and this type we will discuss here. The structure-based study by Li et al. was one of the pioneering studies in the development of NIK-inhibiting compounds (70). By using a small-molecule compound library based on the inhibition of a truncated form of NIK containing only the catalytic domain, they identified imidazopyridinyl pyrimidinamine 1, which laid the foundation of the development of more potent and selective NIK inhibitors (**Table 1**). Next, Demchenko et al. published the novel Amgen compound AM-0216 which is now commercially available as Amgen16 (71). The authors demonstrated inhibition of non-canonical NF-κB signaling in NIK-dependent MM cell lines whereas MM cell lines containing mutations that activate non-canonical NF-κB signaling independent of NIK, were not affected. Inhibition of p52 nuclear translocation upon *in vitro* Amgen16 treatment was later also demonstrated in MCL and another study in the context of inflammation (72, 73). However, the poor pharmacokinetic properties of this inhibitor prevented further *in vivo* studies. In support, other studies also report poor bioavailability *in vivo* and a short metabolic half-life of similar NIK-inhibiting compounds (78, 79). Another structure-based study identified a new NIK-inhibiting compound that was able to specifically inhibit nuclear translocation of non-canonical NF-κB while maintaining canonical NF-κB translocation (74). A novel compound screening study by Pippione et al. identified the NIK inhibitor aminopyrazole 3a (75). Though its specificity remains partly unclear, the authors showed efficacy in a NIK-dependent MM cell line using a general NF-κB gene reporter assay readout, with no significant inhibition in two NIK-independent MM cell lines. One year later, a new NIK-targeting compound was identified by Cheng et al. with a relatively high IC50 value (76). It would be interesting to test this inhibitor in a B cell malignancy model for more accurate comparison. Finally, we have recently published the novel NIK inhibitor CW15337 and applied it to primary CLL cells (77). We showed that lower concentrations of up to 0.25µM CW15337 resulted in specific non-canonical NF-κB inhibition without an effect on cell viability. Higher concentrations of the inhibitor resulted in a full blockade of non-canonical NF-κB signaling along with partial inhibition of canonical NF-κB signaling. Furthermore, we demonstrated that NIK inhibition in CLL cells abrogated CD40-mediated drug resistance and may sensitize CLL cells to venetoclax.

**Table 1:** Overview of NIK inhibitors applied *in vitro*.

Inhibitor	Structure	Model	Cells	IC50	Reference
Imidazopyridinyl pyrimidinamine 1	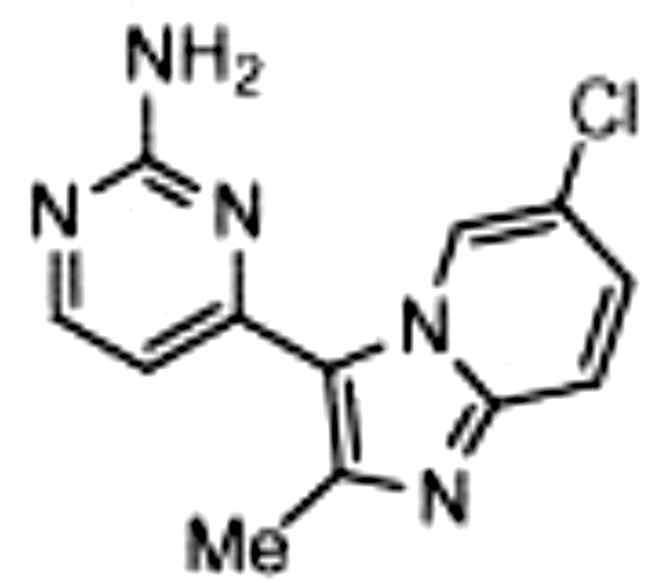	Colorectal adenocarcinoma	HT-29 cell line	16 µM	70
Amgen16	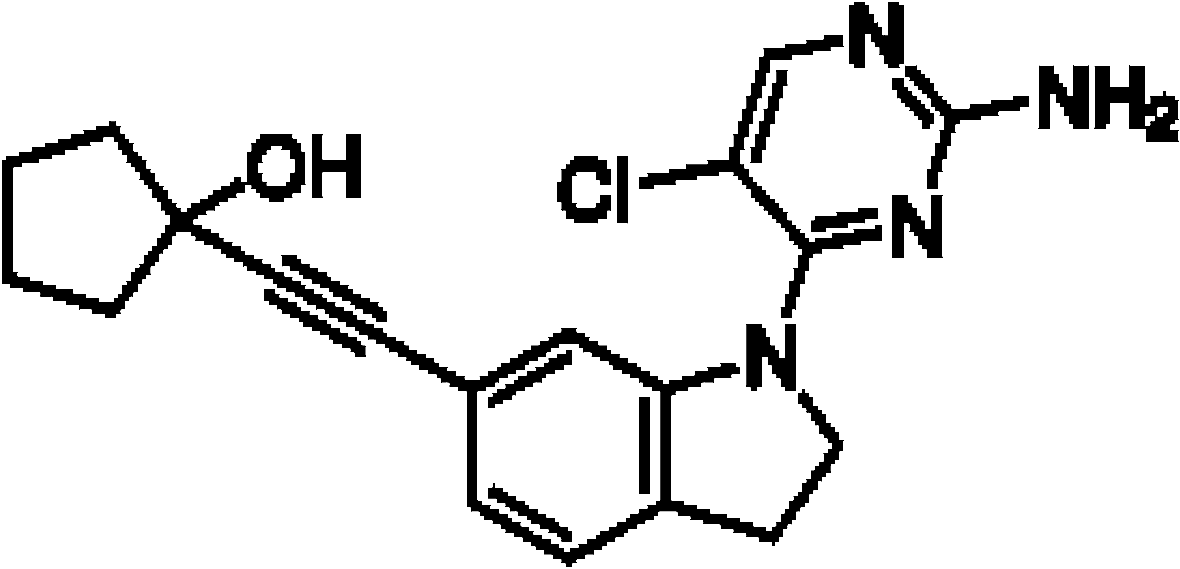	Multiple myeloma Mantle cell lymphoma Osteosarcoma	L363, KMS11 and JMW1 cell lines Z138 and MAVER-1 cell lines U-2 OS cell line	0.417-2.517 µM	71 72 73
Compound 10	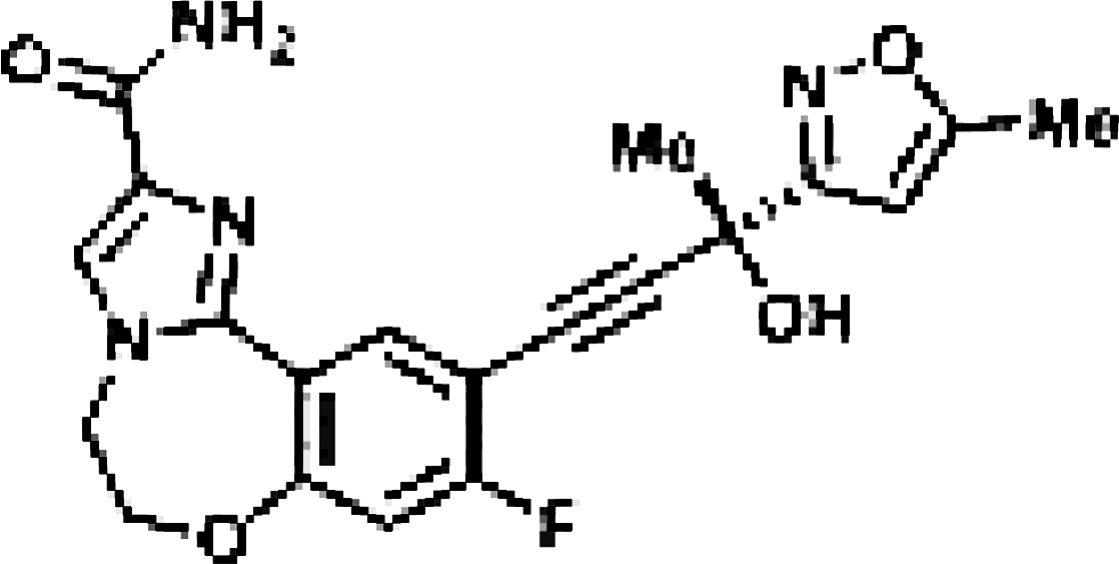	Cervical cancer	HEK293 cell line	123-189 nM	74
Aminopyrazole 3a	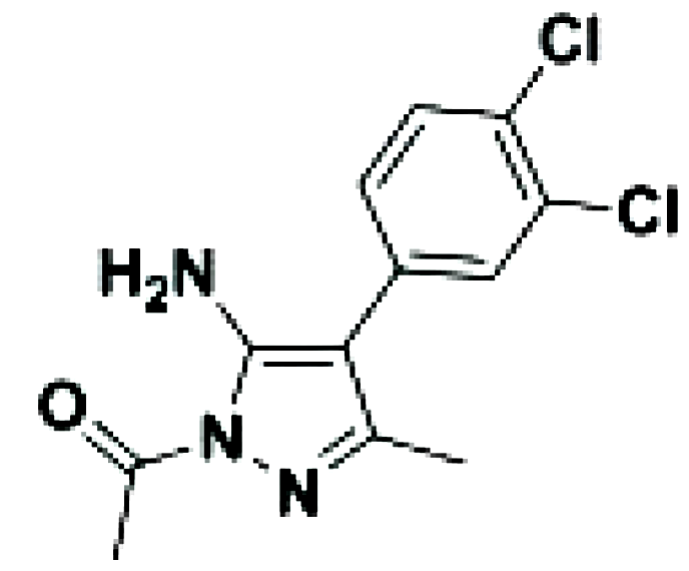	Multiple myeloma	EJM cell line	7.1-9.7 µM	75
N-(3-(6-benzamido-3a,7a-dihydrobenzo[d]oxazol-2-yl)-phenyl)benzamide	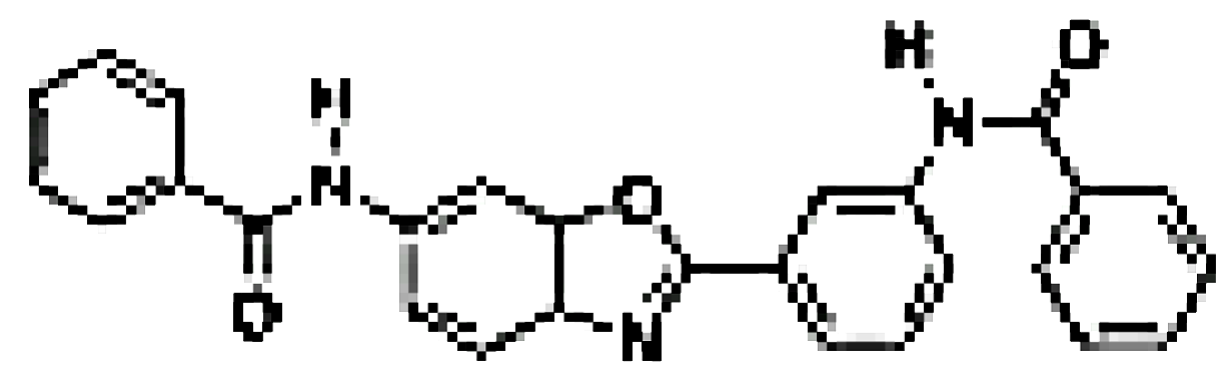	Pancreatic cancer	SW1990 cell line	42-55.8 µM	76
CW15337	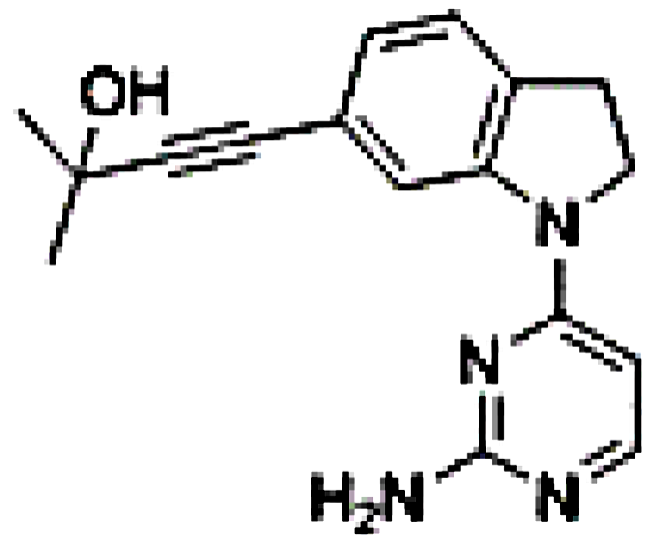	Chronic lymphocytic leukemia	Primary cells	0.5-1 µM	77

## Overview of *In Vivo* NIK Inhibitors

Next, we will discuss some NIK inhibitors that have also been applied in *in vivo* models. Due to the lack of *in vivo* NIK inhibition in the context of B cell malignancies, we will discuss some alternative mouse models, which allow us to learn about the efficacy and toxicities of these NIK inhibitors (**Table 2**).

**Table 2:** Overview of NIK inhibitors applied *in vivo*.

Inhibitor	Structure	Model	Mice	IC50	Reference
B022	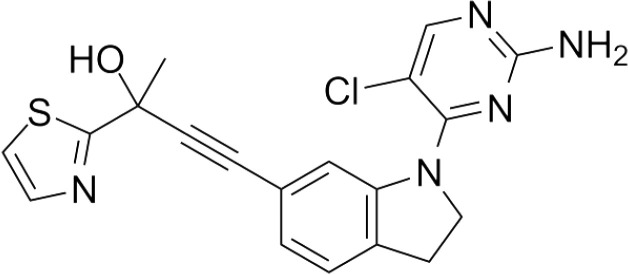	Liver injury & fibrosis Diabetes Alcoholic liver disease	Liver-restricted NIK overexpression β cell-restricted NIK overexpression WT mice on ethanol diet	Unknown	80 81 82
NIK SMI1	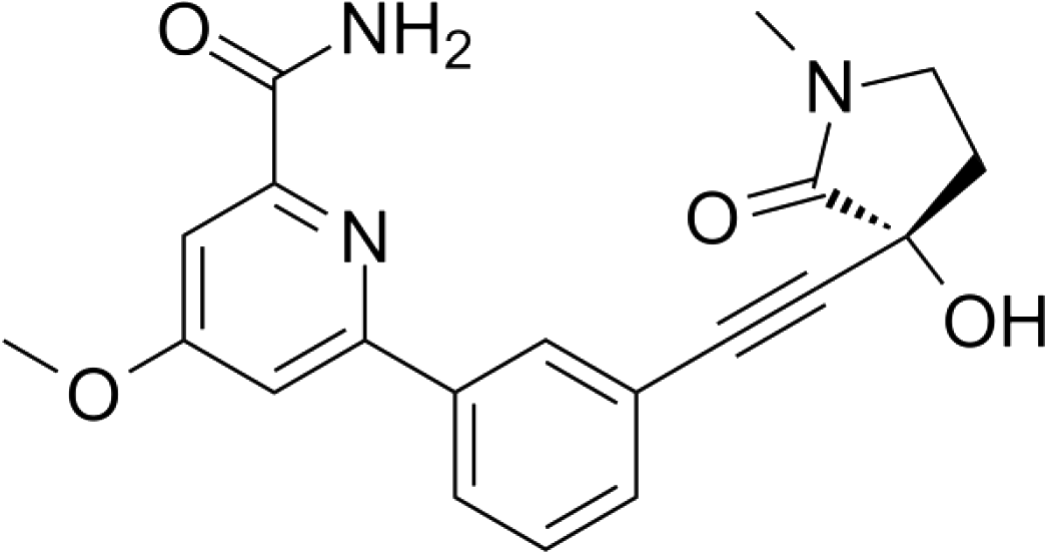	Systemic lupus erythematosus	WT mice, NZB/W F1 lupus mice Intrahepatic cholangiocarcinoma xenografts	108.6-269.4 nM	83 84
Compound 4f	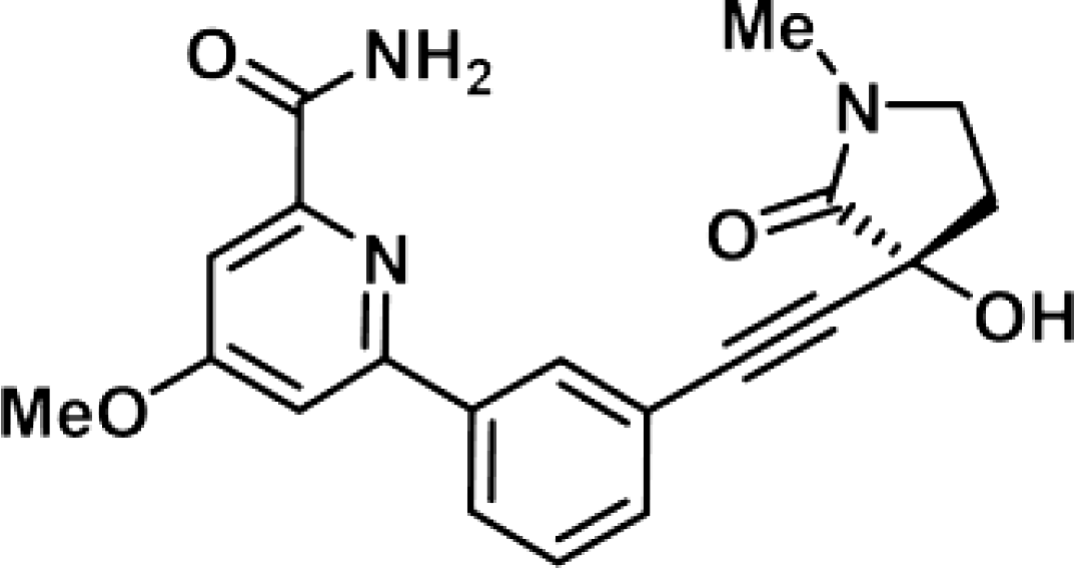		WT mice	309-437 nM	85

The commercially available NIK inhibitor B022, which is structurally very similar to Amgen16, was the first small molecule inhibitor targeting NIK to be applied in *in vivo* models (80). Using this inhibitor, Ren et al. showed inhibition of p100-to-p52 processing and utilized a liver-specific NIK overexpression mouse model. Intravenous B022 administration completely abrogated death as a result of liver inflammation and injury, though general toxicities remain unclear. The authors do note that, just like Amgen16 (71) and in line with another study in diabetic mice (81), due to the bad *in vivo* pharmacokinetic properties of B022, further modifications are needed to achieve efficient long-term *in vivo* NIK inhibition. B022 has also been used to treat mice in the context of alcoholic liver disease, where it alleviated alcoholic steatosis, yet toxicities remain unclear (82). Finally, *in vitro* B022 treatment of mouse stem cells prevented adipogenesis and inhibited non-canonical NF-κB signaling (86). Although these cells do not show nuclear canonical NF-κB, B022 treatment did reduce cytoplasmic RelA phosphorylation levels.

NIK SMI1 is another commercially available small molecule inhibitor of NIK that was generated and published by Brightbill et al. (83). The authors demonstrate an *in vitro* IC50 value in the nanomolar range in primary B cells with a high selectivity for non-canonical NF-κB signaling as RelA activity was not affected. NKI SMI1 administration to WT mice also indicated a dose-dependent effect on B cell survival with a reduction of marginal zone and follicular B cells as well as reduced serum IgA levels, consistent with the phenotype of NIK-deficient mice (53). Consistently, NIK inhibition suppressed *in vivo* immune responses based on reduced germinal center development and IgG1 production, as well as effector/memory T cell generation. NIK SMI1 treatment of a systemic lupus erythematosus mouse model with an autoantibody response inhibited germinal center B cell and plasma cell differentiation, while maintaining normal splenocyte counts. The same was true for the differentiation of CD4 effector memory and follicular helper T cells, without changes in total CD4 T cell numbers. Finally, NIK SMI1 treatment significantly reduced mortality, demonstrating a clear therapeutic effect in hyperinflammatory diseases. In the same year of 2018, Blaquiere et al. published a compound screen where they selected a compound with a favorable *in vivo* pharmacokinetic profile and a molecular structure that is almost identical to NIK SMI1 (85). Consistent with the study of Brightbill et al., the authors found a dose-dependent inhibition of B cell survival *in vitro* and *in vivo*. In mice, NIK inhibition significantly reduced the number of marginal zone B cells, similar to BAFF blockade. A recent study using mouse intrahepatic cholangiocarcinoma xenografts reported good tolerance of NIK SMI1 without weight loss or changes in blood test results (84). They reported an inhibition of tumor cell proliferation without apoptosis both *in vivo* and *in vitro*, pointing towards clinical relevance, potentially in combination with cytotoxic agents.

## Discussion

We have highlighted the importance and advances in the development of NIK inhibitors. The increasing number of studies focusing on the development of compounds targeting NIK may bring NIK closer to the clinic as a therapeutic candidate. However, there are relatively little studies investigating NIK inhibition *in vitro* and *in vivo* in an applied setting. *In vitro* studies and compound screens are mostly performed in standard cell lines whereas *in vivo* studies are mostly outside the context of malignancy. Notably, most of the published studies applying NIK inhibitors lack data on the specificity of the applied NIK inhibitors, and should show the effects of NIK inhibition on both canonical and non-canonical NF-κB pathways, including off-target effects. Importantly, we still lack clinical information on the off-target effects, safety and efficacy of NIK-targeting drugs *in vivo*. Altogether, the investigation of NIK inhibitors has been relatively underexplored, and to date, no specific NIK nor other NF-κB inhibitor has been clinically approved. Existing concerns about toxicities as a result of the broad spectrum of physiological roles of NF-κB need to be further resolved and likely contribute to the fact that none of the NIK inhibitors developed by various pharmaceutical companies has entered clinical trials. Regarding potential adverse effects of NIK inhibition in patients, the best guide is provided by human genetic NIK deficiency. Specifically in the context of B cell malignancies, the requirement for NIK in hematopoiesis may pose potential risks for patients (87). Although the impact of *de novo* NIK targeting may differ from NIK deficiency during development, kinase inhibitors will likely have off-target effects as well. In addition to inhibition of key components of the non-canonical NF-κB pathway, an alternative clinically relevant approach is to block downstream targets or upstream stimulators, or to target crosstalk mechanisms with parallel signaling networks in order to limit toxicities. Due to the many roles of NIK and non-canonical NF-κB signaling in immunity, long-term application of a NIK inhibitor could result in immunodeficiency. Therefore, NIK inhibition in therapy should preferably be used in shorter periods of time (88). While currently only hypothetical, time-limited application of NIK inhibitors may complement existing therapies in the future (77). In conclusion, despite NIK being a promising therapeutic target in many B cell malignancies, current studies on NIK inhibition lack information on specificity, toxicities as well as data on efficacy in the context of malignancy.

## Author Contributions

MH performed the literature study, designed the article, and drafted the manuscript. EE reviewed and edited the manuscript. All authors contributed to the article and approved the submitted version.

## Funding

This work was funded by the Blaauboer Fund via the AMC foundation.

## Conflict of Interest

The authors declare that the research was conducted in the absence of any commercial or financial relationships that could be construed as a potential conflict of interest.

## Publisher’s Note

All claims expressed in this article are solely those of the authors and do not necessarily represent those of their affiliated organizations, or those of the publisher, the editors and the reviewers. Any product that may be evaluated in this article, or claim that may be made by its manufacturer, is not guaranteed or endorsed by the publisher.
